# Gender-specific anatomical correlations of schizotypy in healthy individuals

**DOI:** 10.1192/j.eurpsy.2023.1932

**Published:** 2023-07-19

**Authors:** A. S. Tomyshev, Y. Panikratova, I. Lebedeva, E. Abdullina, E. Pechenkova

**Affiliations:** 1 Mental Health Research Center; 2 HSE University, Moscow, Russian Federation

## Abstract

**Introduction:**

Schizotypy refers to a continuum of symptoms from subclinical manifestations in the general population to severe symptoms in schizophrenia spectrum disorders. Neuroimaging studies revealed significant relationships between schizotypy and cortical anatomy in the general population. However, it remains unclear whether these structural associations has a gender specificity.

**Objectives:**

The present study used structural MRI data to investigate the relationship between subclinical schizotypy symptoms and cortical and subcortical morphometric measures in male and female samples of healthy individuals.

**Methods:**

164 right-handed healthy unmedicated individuals (18.0-34.9 years, 57% females) underwent structural MRI at 3T Philips scanner. T1-weighted images were processed via FreeSurfer 6.0 to quantify cortical thickness for 34 regions-of-interest (ROIs) according to Desikan atlas and volumes for 7 subcortical structures at each hemisphere. Schizotypy levels were assessed using self-report Schizotypal Personality Questionnaire, total schizotypy score and 4 factors scores (Cognitive-perceptual, negative, disorganized and paranoid factors as per Stefanis *et al*. Schizophr Bull. 2004; 30 335-350) were calculated. Partial correlation analysis (ppcor version 1.1, R version 4.2.1) was used to assess the associations between ROIs cortical thickness and total schizotypy or 4 factors scores including age and sex as covariates. The same analysis was performed for subcortical volumes including intracranial volume as additional covariate.

**Results:**

In male group we revealed a positive correlation between greater thickness of the left caudal middle frontal gyrus and higher total schizotypy (r=0.42, p_unc_=0.0003, 95% CI [0.21–0.60]) and negative factor of schizotypy (r=0.49, p_unc_<0.0001, 95% CI [0.28–0.65]) (Image). No correlations survived correction for multiple comparisons in female sample. There were no differences in age, caudal middle frontal gyrus thickness, total schizotypy or negative factor of schizotypy scores between male and female subgroups.

**Image:**

**Figure:**
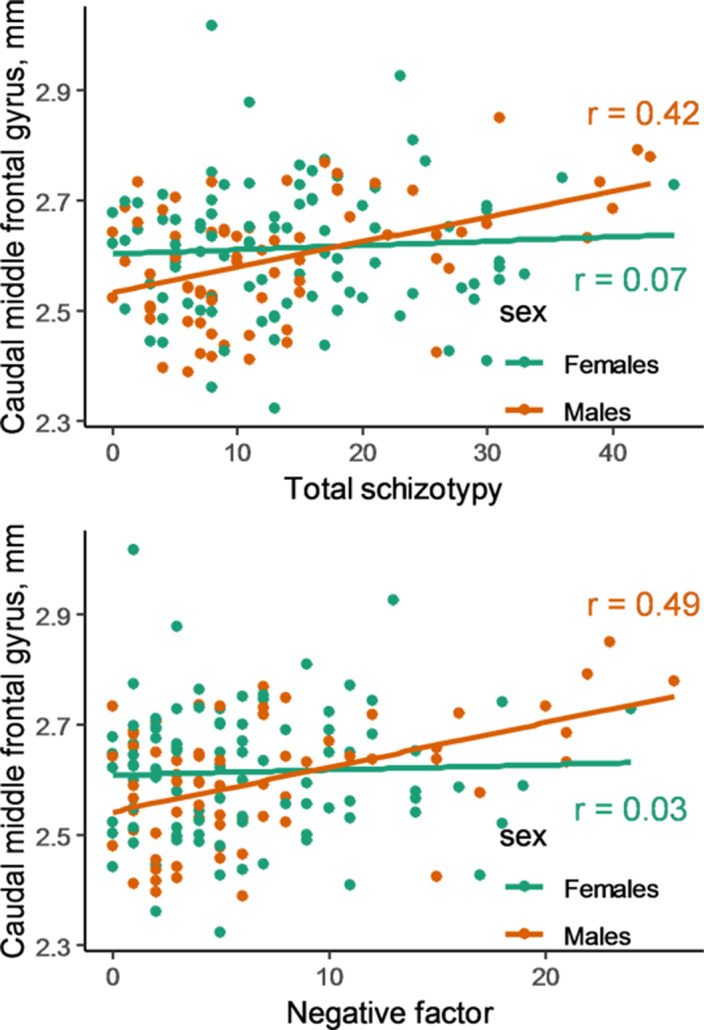

**Conclusions:**

The results suggest that the association of dorsolateral prefrontal cortex (DLPFC) and levels of schizotypy is gender specific. We showed that total and negative schizotypy positively correlated with thicker DLPFC in male but not in female sample. The present data are inverse to findings of prefrontal cortical thinning observed in schizophrenia. Such correlations suggest that thicker cortex could be a potential compensatory mechanism or could reflect alterations in trajectory of cortical thickness reductions across the lifespan.

*The work was supported by RFBR grant 20-013-00748*

**Disclosure of Interest:**

None Declared

